# A Review of Mixed-Effects Models of Tumor Growth and Effects of Anticancer Drug Treatment Used in Population Analysis

**DOI:** 10.1038/psp.2014.12

**Published:** 2014-05-07

**Authors:** B Ribba, N H Holford, P Magni, I Trocóniz, I Gueorguieva, P Girard, C Sarr, M Elishmereni, C Kloft, L E Friberg

**Affiliations:** 1 INRIA, Project-Team NUMED, École Normale Supérieure de Lyon, Lyon, France; 2 Department of Pharmaceutical Biosciences, Uppsala University, Uppsala, Sweden; 3 Dipartimento di Ingegneria Industriale e dell'Informazione, Università degli Studi di Pavia, Pavia, Italy; 4 Department of Pharmacy and Pharmaceutical Technology, School of Pharmacy, University of Navarra, Pamplona, Spain; 5 Global PK/PD Department, Lilly Research Laboratories, Surrey, UK; 6 Merck Institute for Pharmacometrics, EPFL, Lausanne, Switzerland; 7 Advanced Quantitative Sciences Department, Novartis Pharma AG, Basel, Switzerland; 8 Optimata Ltd., Bene Ataroth, Israel; 9 Department of Clinical Pharmacy and Biochemistry, Institute of Pharmacy, Freie Universitaet Berlin, Berlin, Germany

## Abstract

Population modeling of tumor size dynamics has recently emerged as an important tool in pharmacometric research. A series of new mixed-effects models have been reported recently, and we present herein a synthetic view of models with published mathematical equations aimed at describing the dynamics of tumor size in cancer patients following anticancer drug treatment. This selection of models will constitute the basis for the Drug Disease Model Resources (DDMoRe) repository for models on oncology.

## Rationale For a Library of Models and Specificity of the Presented Selection

As the use of modeling and simulation in oncology drug development is becoming more prevalent, there is a need to create a library of models that researchers in pharmaceutical industries and academia can rely on while performing oncology data analysis.

Such a library should offer a unique and standardized platform for storing published mathematical structures of cancer and anticancer drug models, which can serve as a foundation for the analysis of new data sets and for the development of new models. In addition to storing the equations of the structural models, the repository should also store information on the statistical components of each model, in addition to the parameter values and uncertainties. It can then be used to retrieve a model that adequately captures a given biological system, to obtain initial estimates, or to compare final estimates of different models. The preliminary version of the library will not only include models for tumor growth data and efficacy data of anticancer drugs but also models for toxicity data and models for circulating biomarkers and overall survival in the context of anticancer drug development and use in clinical practice. Herein, we report mixed-effect models of growth and effects of anticancer drug treatment. We focused our selection on models applied in a population analysis context, given the solid value of this statistical approach in the integration of different levels of variability inherent to any biological process. As a consequence, this review is not exhaustive. In particular, more mechanistic and biologically and pharmacologically plausible models—limited nowadays by the availability of data and the lack of appropriate statistical tools for parameter estimation and model evaluation, but with clear potential to tackle the critical challenges facing early drug development in oncology, e.g., exploring effects at different target sites—will not be discussed here. The reader can find further information and other views of oncology models in recent reviews^1,2,3^ and in the book by Bonate.^4^

## First Attempts to Model Tumor Growth

Very early attempts to characterize tumor dynamics were generally based on the belief that the process of tumor growth follows a simple exponential model.^5^ The hypothesis of an exponential growth process was in accordance with the idea that, under ideal conditions—i.e., all tumor cells have sufficient nutrients and oxygen—all cells composing the colony should proliferate, leading after mitosis of one tumor cell to eventually two new ones.

However, starting from 1930, researchers began to observe that the diameters of grafted sarcomas in rats increased linearly with time (*t*), or that, in other words, the tumor volume (*V*) increased according to a cubic law^6^: 
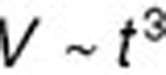
. Following the observation of a deceleration of the growth rate, without any phase at which the growth rate remained constant, the Gompertz equation^7^ was introduced for the first time in the context of tumor growth analysis and was shown to describe the growth of different types of tumors in animal models.^5^

Laird, in ref. 5, formulated the Gompertz model of tumor size (or number of cells) *y* over time *t* as follows:

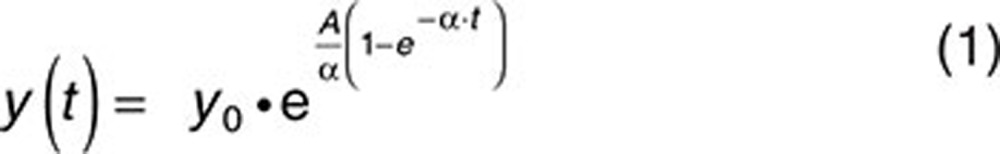


where *y*_0_ denotes the tumor size (or number of cells) at time 0, and *A* and α are two positive constants regulating both growth rate and saturation size. Specifically, *A* is the initial growth rate of the process, and α stands for the deceleration rate related to the natural death of the tumor cells. The model can also be written as a system of ordinary differential equations, which allows for a better understanding of the two simultaneous processes that occur: exponential growth with a nonconstant growth rate (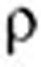) that decelerates exponentially as the tumor grows:

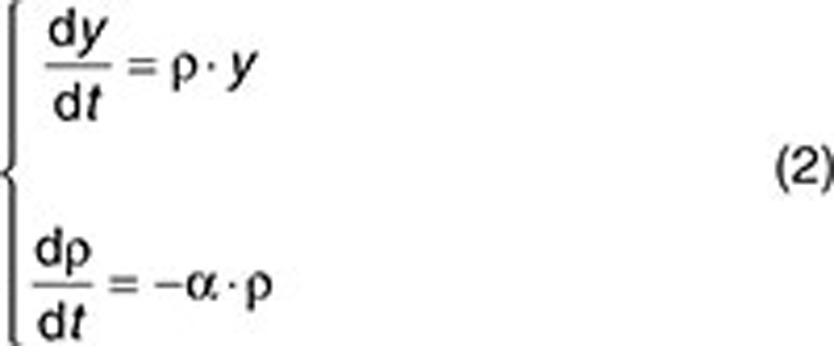


Eq. 1 is a solution of system represented in Eq. 2 if the initial value of 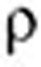 is assumed to be equal to *A*.

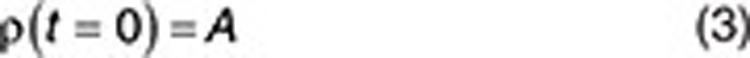


Note that here, the deceleration of the growth rate of the tumor is assumed to be “physiological,” i.e., to be associated with the natural death of tumor cells, a process that is dependent on the present tumor volume (number of tumor cells).

The Gompertz equation can also be formulated as follows:

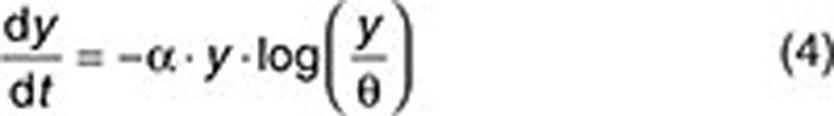


where Θ denotes the maximal size that *y* can reach. It can be shown that this Eq. 4 is equivalent to the two previous ones (i.e., Eqs. 2 and 3) when

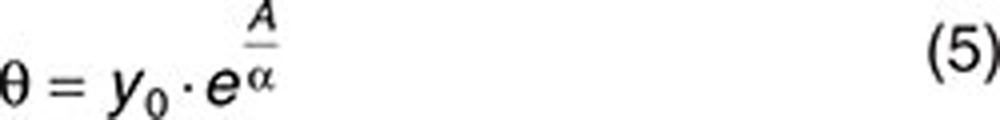


Biologically speaking, the Gompertz model offers several advantages relative to the exponential growth model. In particular, the Gompertz model captures, within a continuous process, the tumor cells' dependence on the availability of nutrients, oxygen, and space. As the tumor grows, the availability of these vital resources gradually decreases, leading to a deceleration of the growth rate, until the tumor size attains its maximum value (saturation threshold), denoted Θ.

After being successfully used to describe the dynamics of tumor size in animal experiments, the Gompertz model was first used in human patients to describe tumor growth in multiple myeloma.^8^ On the basis of *in vivo* and *in vitro* assessments of the rate of production of an immunoglobulin marker, the Gompertz model was shown to fit the observations of the dynamics of myeloma cell number over time.

The Gompertz model was also used to analyze survival data reported in ref. 9 for 250 female patients with untreated breast cancer who were hospitalized between 1805 and 1933 at the Middlesex Hospital in London.^10^ Although tumor size measurements were not available, the researchers simulated the Gompertz model using tumor growth parameter values within a feasible range, and they calculated how long it would take until the tumor size became lethal to the patient (a “death” size threshold). A survival curve was generated and compared with the observed survival data.

## Tumor Growth Models Used as a Tool to Better Evaluate Treatment Response

In solid-tumor clinical trials, tumor size is typically measured using imaging techniques (e.g., computed tomography scan, X-ray) and recorded according to the Response Evaluation Criteria in Solid Tumors (RECIST)^11^ as the sum of the longest diameters across targeted lesions measured on a limited number of organs. Then, according to RECIST, tumor size is transformed into an objective tumor response by classifying the continuous measurement according to one of four categories, namely, complete response, partial response, stable disease, and progressive disease. This step facilitates clinical interpretation of the measurements. This assessment method of drug effectiveness/resistance based on a point estimate, i.e., best overall response, has its limitations.^12,13^ First, the transformation of a continuous variable into a four-category variable results in the loss of a great deal of information; second, the RECIST criteria are evaluated at discrete time points, and all the dynamic characteristics of tumor growth, treatment-related shrinkage, and resistance development are ignored. As a result, the use of RECIST to evaluate dose response—one of the key objectives of early drug development—is very challenging.

Since 2004, the US Food and Drug Administration, through its Critical Path Initiative, has been promoting quantitative modeling to improve the quality of decision making in the drug development process.^14^ In line with the Critical Path Initiative, recent articles have presented new and innovative ways of leveraging the available RECIST clinical data in order to improve assessment of drug efficacy/resistance in the early to middle stages of drug development. Several models have been proposed to describe the time course of tumor size, expressed as the sum of the longest tumor dimensions, as opposed to the four categories of “objective tumor response.” These models use mathematical expressions ranging from simple analytical expressions to complex mathematical systems written with ordinary differential equations.

From 2008 up to now, 13 published papers have proposed an analysis of the time course of tumor size in patients in eight different therapeutic indications: colorectal cancer,^15,16^ non–small cell lung cancer,^17,18^ renal cell carcinoma,^19,20,21,22^ thyroid cancer,^23^ metastatic breast cancer,^24^ prostate cancer,^25^ gastrointestinal stromal tumor,^19,26^ and low-grade glioma.^27^ In the next section, we provide a comprehensive description and synthesis of the mathematical expressions used in these models.

## Models Expressed as Algebraic Equations

Several different studies have used algebraic equations to describe tumor size dynamics. In ref. 18, the tumor size curve over time is described as a combination of linear growth and exponential decay. The model can be written as follows:

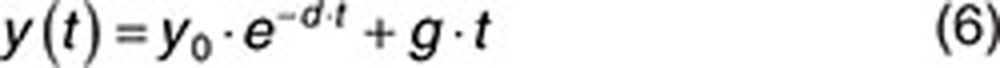


where *y* is the predicted sum of the longest tumor diameters, and *t* represents the time elapsed since the beginning of the observation period and also corresponds to the time of treatment onset. The parameter *y*_0_ is the tumor size at the first measurement for the patient (also called the baseline tumor size), and *d* and *g* are, respectively, the drug-induced decay and net growth parameters. The model was applied to data collected in 3,398 non–small cell lung cancer patients receiving either mono- or polychemotherapy or placebo treatment.^18^ Note that *d* relates to the drug/placebo effect but has not been explicitly linked to actual doses or concentrations in this publication.

Stein *et al*. in ref. 25, proposed the following expression:

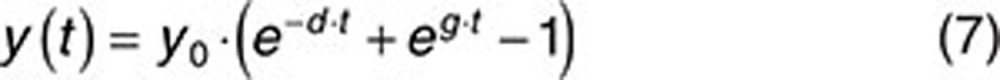


The term “—1” is used to ensure that at time 0, tumor size *y*(0) *= y*_0_.

Here, tumor size is assumed to increase and decrease exponentially, with *d* and *g* being the rate constants for the drug-induced decay and the net growth of the tumor size, respectively. Note that in the original article, this formula, even if proposed for the analysis of tumor size measurements, was applied to the analysis of the time course of prostate-specific antigen data. However, the same model was successfully used in ref. 16 to analyze tumor size dynamics in 1,126 patients with metastatic colorectal cancer. As in ref. 18, parameters were estimated by matching *y* to tumor size measurements from patients, calculated according to the longest tumor diameters.

Bonate and Suttle in ref. 28 proposed a modification to the model in Eq. 6 with the goal of ensuring a smooth curvature at the nadir tumor size (the transition between decay and growth). Specifically, they added a quadratic growth term:

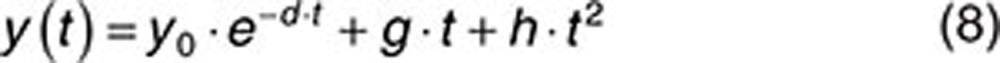


Analytical models present some advantages for implementation: because of their mathematical simplicity, they are easy to implement in classical statistical software programs such as SAS, and computations are very quick. However, they have several disadvantages. First, they cannot account for varying dosing information (e.g., dose deescalation and modification). Second, they are purely empirical in nature and therefore cannot be used to extrapolate tumor dynamics to different dosing regimens or even to account for changes in a dosing regimen within a study. And finally, they are of limited use in attempts to understand or formulate new hypotheses regarding the mechanisms driving tumor growth and response to treatments.

## Models Expressed as Ordinary Differential Equations

### Models for the analysis of clinical data

Ordinary differential equations are used to describe elementary changes in tumor size as a function of net growth (i.e., the difference between growth and natural death) and drug-induced decay processes. Generally, this family of models can be written as:

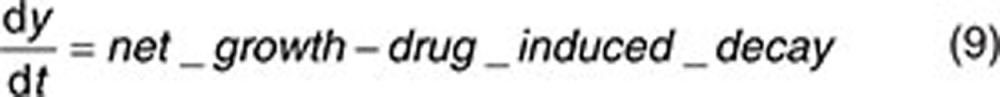


where 
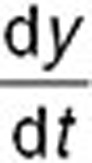
 denotes the derivative in time of the tumor size, i.e., the change in tumor size over time.

The growth captured in *net_growth* can take different forms, including, e.g., linear growth (*net_growth* equal to *a*, with *a* being a constant), exponential growth (*net_growth* equal to 
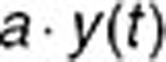
, with *a* being a constant), or more complex forms that separate growth and natural decay (not induced by drugs).

It is useful to introduce a general growth law, called a “generalized logistic” law, which can be written as follows:

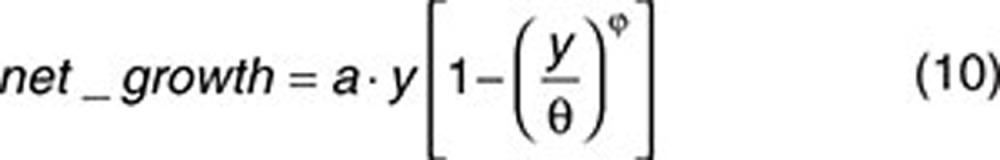


with *a*, Θ and 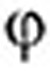 as three positive rate constants. Note that this expression can be interpreted as growth (
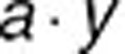
) complemented by natural tumor cell death 
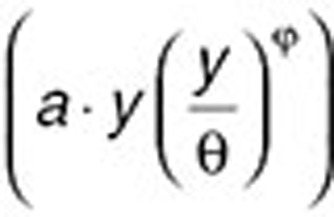
, which induces saturation.

In the Gompertz model, the parameter Θ denotes a maximal size above which the tumor will not be able to grow. If 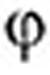 is equal to one, Eq. 10 becomes the “logistic model”:

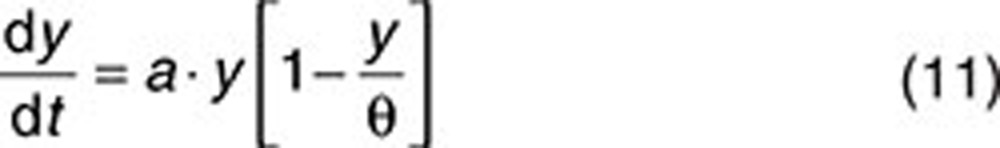


If, on the other hand, parameter 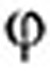 tends to zero, resulting in slow deceleration of the net growth rate (e.g., 
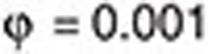
 ), Eq. 10 is an approximation of the Gompertz model as written in Eq. 4 because 
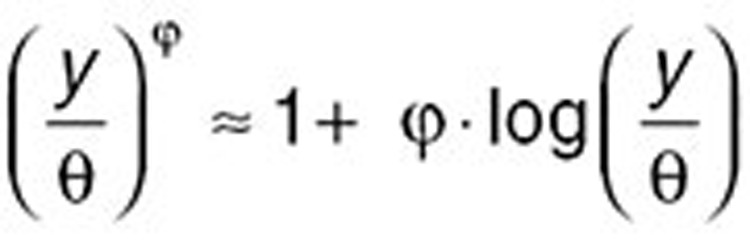
 when 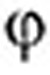 is small. The parameter 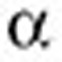 from Eq. 4 is then equal to 
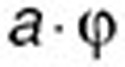
.

The drug-induced decay term in Eq. 9 can represent the action of an anticancer drug and is generally assumed to represent a first-order decay process as follows:

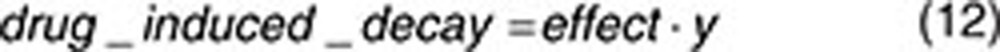


As a practical comment, this insures nonnegativity of the solution for *y*(*t*).

The term *effect* can represent a constant parameter or a function of a metric of drug exposure (e.g., plasma concentration), e.g.,

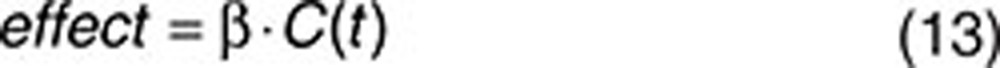


where *C(t)* denotes the drug concentration, either in plasma or at the effect site. Note that *effect* can also be related nonlinearly to *C(t)*, e.g., through an *E*_max_ model. The term β can be either constant or time dependent, as in the following example:

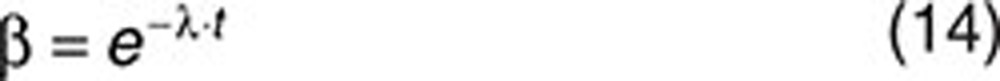


In such a way, the decay term decreases with time. This expression accounts for the loss in *drug_induced_decay* over time due to declining efficacy of the drug, e.g., because of the emergence of “resistance.” A plausible biological explanation for such resistance is heterogeneous drug sensitivity of cells within the tumor mass. Another explanation could be the possible emergence, with time, of cell mutations conferring properties of resistance to the drug. Note that β can also be dependent on other terms, including, e.g., the time course of drug concentration.

**Figure 1:**
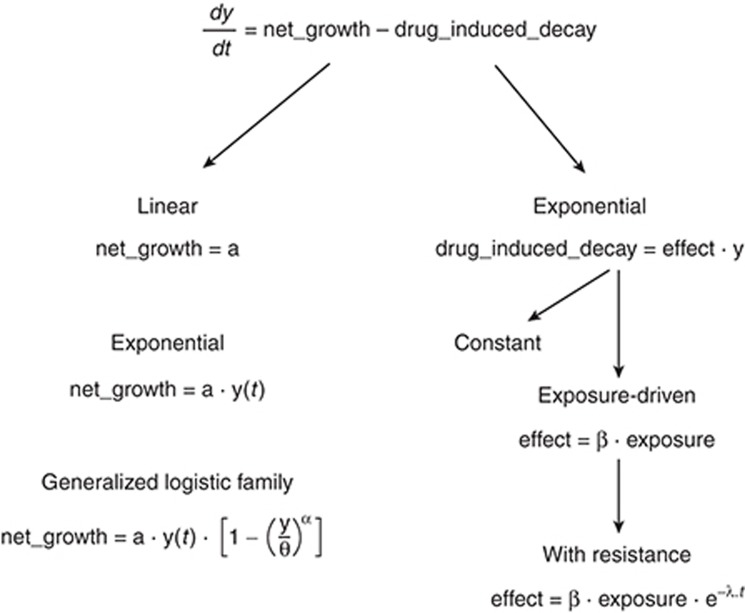
General differential model for tumor size dynamics and development of the growth and decay terms as encountered in the reported publications.

**Figure 1** proposes a summary of the different terms for *net_growth* and *drug_induced_decay* in the general differential equation model for tumor size dynamics.

Among the 13 published studies mentioned above, almost two thirds (8/13) present a model based on differential equations.

**Table 1** shows a summary of the various main models together with their associated mathematical expressions.

The model proposed in ref. 27 and applied to tumor size data in low-grade glioma patients is the most complex so far. In particular, the model is formulated as a system of ordinary differential equations that incorporate different types of tumor tissues. This formulation is supported by the fact that these tumors are known to be composed primarily of nonproliferative quiescent tumor cells.^30^ Despite this complexity, the same logic governs the balance between net growth and drug-induced decay processes within the different tumor cell compartments (i.e., the different types of tissues). In particular, the growth term of the proliferative cell compartment is modeled using a logistic term.

Finally, the model presented in ref. 17 uses a logistic term (Eq. 11) for the growth component of tumor size progression, in which the tumor saturation size (i.e., the maximal size it can attain) is the baseline size, *y*_*0*_. This means that after the shrinking effect of the drug diminishes entirely, the tumor will be able to grow until its baseline value. The drug action is taken into account with an “*E*_max_” model, formulated such that the effect of the drug is to reduce the growth rate of the tumor. The model can be written as follows:

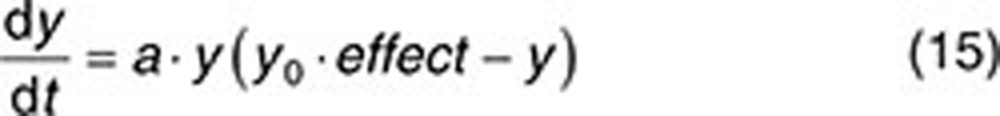


where 
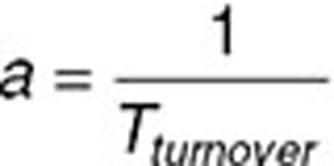
 following the notation of the publication^17^ and 
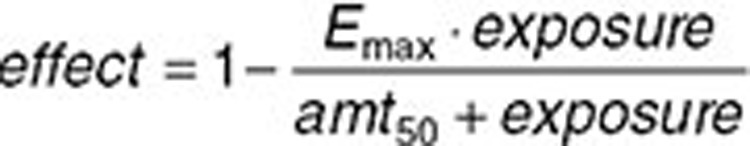
. This model incorporates the drug-induced decay term differently compared with the general model proposed in Eq. 9. In fact, it is the only model for clinical data developed thus far in which the drug is assumed to decay the growth rate and not to induce a direct reduction of the tumor size. The authors chose this modeling strategy because the drug compound they modeled was known to act by slowing tumor cells' growth, through its influence on their DNA, rather than by increasing the death rate of surviving cells. In the analysis of preclinical data, a similar strategy has been chosen to model the action of antiangiogenic drugs, whose primary mode of action consists of inhibiting tumor growth through the disruption of the formation of intratumoral blood vessels.^31,32,33^

It is important to note that the development and analysis of each model presented here were carried out using a population (mixed-effects) approach, with the exception of the analysis of prostate-specific antigen dynamics in ref. 25. The patient sample sizes in the various models ranged from 56 (ref. 17) to 3,398 (ref. 18), with a median of 391 individuals. Regarding parameter estimation, the algebraic models are the least complex, incorporating only three structural parameters.^16,18,21,25^ The other models each include four structural parameters, with the exception of the model in ref. 27, which used eight structural parameters.

In 12 of the 13 studies discussed above, the model variable *y*(*t*) was matched to the observed sum of the longest dimensions of the tumor. The one exception was that of the study on low-grade glioma^27^; this model used mean tumor diameter as the dependent variable. This choice was in agreement with a standard approach for treatment evaluation in low-grade gliomas.^34^ Regarding the software used for parameter estimations, all mixed-effects models relied on the NONMEM software,^35^ except for the brain tumor study, where Monolix (Lixoft) was used.^27^

A summary of the 13 studies, presenting analysis of tumor size dynamics in clinical settings, is shown in **Table 2**.

### Models for the analysis of preclinical data

Preclinical experiments comprise an important stage in the process of understanding drug effects. A multitude of different techniques, animals, and processes can be used to carry out relevant experiments, specifically adapted for each drug of interest. In oncology, exploratory preclinical studies often entail s.c. implantation of human tumor fragments into the flanks of nude mice (xenograft). Once the tumors have reached a predetermined size, drugs are administered. Slide calipers are used to periodically measure tumor size over a predefined period of time. A standard measurement approach is to measure three tumor diameters and to calculate the corresponding geometric mean, which is then an approximation of the tumor volume.^40^

In the section that follows, we present several preclinical models whose mathematical representations are similar to those presented in the previous section. One modeling framework often used for the analysis of tumor size dynamics in mice is presented in ref. 38. This framework relies on a general model for time-dependent transduction systems.^39^ Three different models are presented to describe the action of a drug on tumor cells:

1. The first model formulation assumes a first-order (exponential) net growth process with a drug-induced decay term that is a function of the drug concentration and is formulated as an “*E*_max_” model:

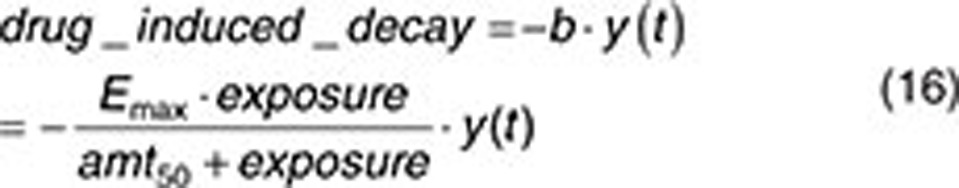
2. The second formulation assumes that the drug acts on a fraction of the tumor cells. A second cell type (or compartment) is thus introduced with back-and-forth exchange with the first compartment (on which the drug is active). The compartments are typically assumed to correlate with phases of cell division, but experimental data are typically not available for modeling to support such an assumption.
3. The third formulation assumes a delay in the action of the drug. Four transit compartments are introduced to delay the effect of the decay term, which represents the action of the compounds. This formulation was introduced to account for delays in drug effect on tumor size, commonly observed in mice treated with chemotherapeutic compounds.

The model proposed in ref. 40 is similar to the “transit compartment” model in ref. 38. In this model, the growth term is modified to account for a transition between an initial exponential growth phase (which is supposed to reflect the initial growth process, when tumor cells have enough oxygen to proliferate) and linear growth (when the availability of nutrients and oxygen becomes limited due to excessive tumor size). Thus, the model is based on a different biological assumption from that of the Gompertz model because a hypothetical switch occurs between the two growth phases. A transit chain is coupled to the affected cells to account for the duration of the death process after drug action. Thus, the transit chain applies to the process of death and not, as in ref. 38, to the process of the drug's distribution to its site of action. On the basis of the model in ref. 40, Shah *et al*. developed a multiscale mechanism–based pharmacokinetic/pharmacodynamic model for antibody–drug conjugates in mice and successfully predicted progression-free survival rates and complete response rates in patients treated with brentuximab–vedotin.^41^ A similar translational strategy was applied by Haddish-Berhane *et al*. to predict first-in-human doses of antibody–drug conjugates.^42^

The model proposed in ref. 31 for evaluating the effect of an antiangiogenic drug in preclinical experiments is similar to the model in ref. 17, which describes the time course of tumor size in non–small cell lung cancer patients treated with gemcitabine. A Gompertz model is used for the net growth process, in which the saturation size (parameter 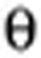 in Eqs. 4, 10, or 11) is not constant but varies as a function of different parameters and, in particular, of a drug effect parameter. Typically, administration of a drug induces a reduction of 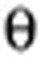. Thus, as in ref. 17, in the model of ref. 31, the drug acts by reducing the growth rate and not directly by inducing decay in the tumor size. A similar idea was explored in ref. 32, where the growth dynamics of the untreated tumor proposed in ref. 40 were revised to take into account the growth rate reduction effect of an antiangiogenic drug. Bueno *et al*., in ref. 43 used a similar approach to model the effect of a type 1 receptor transforming growth factor-β kinase antagonist.

## Discussion and Perspectives

For decades, researchers have turned to models of tumor size data to characterize the dynamics of tumor growth and response to treatment. More recently, mixed-effect models have been proposed as a means of facilitating decisions on whether to move to phase III clinical trials based on tumor size response in phase II trials^18,29^ for evaluating the value of biomarkers in predicting tumor response^26^ and for innovative patient treatments in clinical routine.^28,44^ In this review, we have presented the historical development of tumor growth models and have elaborated on recent published nonlinear mixed-effects mathematical models aimed at analyzing tumor size dynamics. We have proposed a synthetic presentation of these models and provided necessary technical details for their implementation and simulation.

The main focus of this review was to report on 13 recent scientific studies presenting eight different mixed-effect models for the analysis of tumor size dynamics in clinical settings. These studies are summarized in **Table 2**, which can serve as a guide for the initial selection of an appropriate model structure according to the investigated cancer indication. We have shown that despite the apparent heterogeneity in the formulations of the differential-equation-based models, they all originate from the same general mathematical expression (Eq. 9), which comprises a net growth term and a drug-induced decay term.

**Figure 2:**
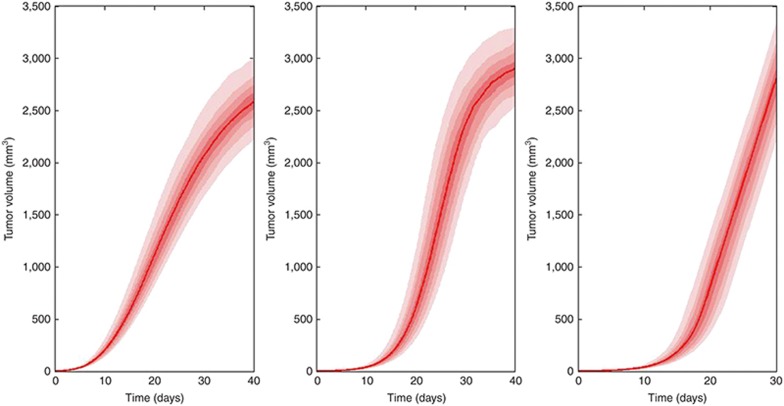
Simulations illustrating plausible growths of tumor volume in xenografted mice for a range of parameter values of the Gompertz (left panel), generalized logistic (middle panel), and Simeoni models^37^ (right panel).

It was shown that the Gompertz equation is at the basis of the development of tumor size models and can be used to account for tumor growth in the absence of treatment. This model includes three parameters (baseline, growth rate, and saturation threshold) and is thus relatively simple, while also being supported by biology (reflecting tumor size limitations due to limited availability of oxygen and nutrients during tumor growth). It also allows for the incorporation of different drug effects and, in particular, reduction of the growth rate through modification of the saturation value. We have seen that the generalized logistic model (Eq. 10) is similar to the Gompertz model with the presence of a saturation value. This model incorporates an additional parameter (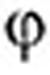 in Eq. 10), which regulates the transition between the initial exponential phase and the growth-saturated phase (a small 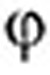 will result in a smooth transition). It should be noted, however, that this model also presents disadvantages. Technically, the saturation size 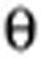 is difficult to estimate based on preclinical or clinical data because the plateau is generally not observed in preclinical or clinical settings. For instance, in both refs. 24 and 27, this parameter was fixed to 10 cm. In ref. 17, it was fixed to the tumor baseline value. **Figure 2** shows simulations for a range of parameter values of the Gompertz, generalized logistic, and Simeoni models^40^ These three classical models rely on similar assumptions, except that the Simeoni model assumes tumor size to grow linearly with time after an initial exponential phase.

Overall, the Gompertz model remains an interesting model for the analysis of tumor size data sets in both preclinical and clinical settings. Of note, however, Hart *et al*., in 1998, showed the better performance of a non–mechanism-based quadratic function of time (i.e., parabolic growth), compared with the Gompertz equation, in the analysis of large-scale breast cancer mammography data.^45^

The rate of drug attrition in oncology has now reached a critical level of 95%, and only 40% of compounds that yield positive results in phase II trials are subsequently successful in phase III trials.^46^ It is thus reasonable to consider that the use of tumor size growth models might facilitate informed, quantitatively based decisions for the drug development process in oncology. One of the main potential benefits of these tools is the possibility, based on early tumor size measurements collected in phase I and phase II trials, to predict the probable efficacy results of phase III clinical trials.^18,29^

The coupling of tumor size models with models describing overall patient survival offers a key new perspective regarding their use. It is also the most likely coupling to succeed. Tumor size data offer a dual advantage: they can be collected early on in clinical development, and they can also serve as a treatment efficacy metric that reflects the ultimate clinical end point (survival) relatively closely. Several studies have shown the potential of modeling tumor size to predict long-term clinical outcomes such as survival. For example, the change in tumor size ratio (TSR) in week 7 after treatment onset has been identified as a predictor of overall survival in colorectal cancer patients treated with capecitabine or 5-fluorouracil.^29^ TSR has since been applied to analyze the effects of several drugs for various tumor types. For example, the tumor size ratio at week 8 after treatment onset was a significant predictor of survival in non–small cell lung cancer^18^ and for thyroid cancer patients treated with motesanib.^23^ Claret *et al*., in ref. 16, proposed using the “time to growth” (time elapsed from the onset of treatment until the tumor reaches its minimal size) as a predictor of overall survival because they found this metric to yield more accurate predictions than those from tumor size ratio in metastatic colon cancer (see also ref. 47) Hansson *et al*.^26,36^ evaluated the full time course of biomarkers, tumor size, and adverse effects for prediction of survival and found these dynamic variables to be superior to constant-value metrics such as the tumor size ratio in a specific week. A recent review presents a summary of metrics that have been incorporated into population models to predict survival in oncology^3^ and provides a discussion of the advantages and disadvantages of each metric.

Such modeling initiatives have aimed at not only better designing of phase III clinical trials but also suggesting innovative methods for providing quantitatively based suggestions to clinicians in treatment of cancer patients in clinical practice. For instance, in ref. 27, the authors suggest that the model can be used to individualize low-grade glioma patient therapy based on the analysis of tumor size data collected before treatment onset. Other models developed under a population context^48,49,50^ or for individual patients are also of use for patient treatment individualization.^49^

Regarding the development of models for preclinical data, efforts are currently under way to develop models that account for the effect of immunotherapy,^51,52,53,54^ models that account for combinations of drugs,^32,52,55^ models to account for intracellular dynamics in combined systems biology/pharmacometric models,^56^ and models to translate preclinical results into clinical findings.^,41,42,57,58^ On this last topic, although recent published studies provide exciting and encouraging results, we think that more complex and plausible models integrating data on biological and pharmacological mechanisms, including biomarkers—together with appropriate parameter estimation and model evaluation methods—will be needed to predict clinical activity from preclinical information. It is also important to carry out data analysis studies based on more sophisticated preclinical models, than the classically used subcutaneous xenografts.

New studies will need to be developed to validate the relevance of these recent initiatives. In this respect, it is hoped that regulatory agencies, together with pharmaceutical companies, will launch a global program to promote the use of such analyses in the investigation of systematically marketed therapies. The European Medicines Agency's Policy 070 on publication and access to clinical trial data will definitively help in that regard by creating the opportunity for scientists to access pools of individual-level data from clinical trials (http://www.ema.europa.eu/docs/en_GB/document_library/Other/2013/06/WC500144730.pdf). Similar initiatives are already available for other therapeutic indications, such as stroke, through the Vista repository (http://www.vista.gla.ac.uk). We believe that access to appropriate data is the most problematic issue for further mechanistic developments and relevant applications of models. In oncology, in addition to the significant costs of evaluating tumor size response through imaging techniques, the method itself of evaluating treatment efficacy in clinical trials with an end point assessed at a specific time point resulted in forgetting the potential interest in evaluating the dynamic aspect of the response. In addition, safety, accessibility, and/or economical aspects also limit the possibility of performing multiple computed tomography scans or magnetic resonance imaging evaluation of tumor size and thereby to fully explore the pharmacokinetic/pharmacodynamic relationships and to characterize natural tumor growth. Finally, the clinical observations used (size of target lesions) could also be discussed; other observations such as new lesion appearance might be of great interest, in particular for extrapolation to survival. The modeling of circulating biomarkers (see refs. 59 and 60 for reviews of modeling initiatives) is also a clear avenue in the development of more mechanistic models and for identification of predictive biomarkers. As an example, the soluble vascular endothelial growth factor receptor has been presented as a longitudinal and mechanism-based predictive biomarker for overall survival following sunitinib treatment in gastrointestinal stromal tumor patients.^26,36^

Another avenue for model improvement is the integration of the underlying biological and pharmacological complexities following a systems pharmacology approach. The development of such mechanistic models may provide insights into the understanding of key features, for instance, the emergence of resistance to treatment. Thus far, the approach used to model drug resistance, and in particular its link to time, has been purely empirical and has been often confounded with the time course of drug elimination. Other approaches linking resistance to either dose or exposure need to be explored. Finally, most models we report here assume drug effects that kill cells directly, whereas the mechanism of action of most drugs is to reduce proliferation and promote apoptosis during mitosis.

Fundamentally, we believe it is important to use biologically and pharmacologically plausible models including integration with modern molecular and systems biology to better describe and represent the underlying biological and pharmacological complexities. The formulation of more mechanistic models, such as those already developed for prostate cancer,^53,61^ chronic myeloid leukemia,^62^ or high-grade glioma,^63,64,65,66^ including models for the analysis of full imaging data, provides an important opportunity. Modeling initiatives will need to follow a multiscale strategy, integrating data and information from the molecular level (molecular pathways targeted by the investigated compounds), through the cellular level (cell cycle regulation), to the macroscopic level (tumor dimensions). Theoretical studies have already proposed methods to link these three integration levels assuming, for instance, the subcellular level dynamics at a steady state when focusing on the timescale of tumor size evolution (see refs. 67,68, as examples). Ongoing technical extensions of mixed-effect regression techniques^69,70^ indicate that it will be soon possible to use these mechanistic models within a population analysis context.

Overall, more concerted efforts by different stakeholders may in the future contribute to further improving the role of modeling and simulation in drug development and for regulatory and therapeutic decisions in oncology.

## Conflict of Interest

The authors declared no conflict of interest.

Deputy Editor-in-Chief Lena Friberg was not involved in the *CPT:PSP* review or decision process for this article.

## Figures and Tables

**Table 1:**
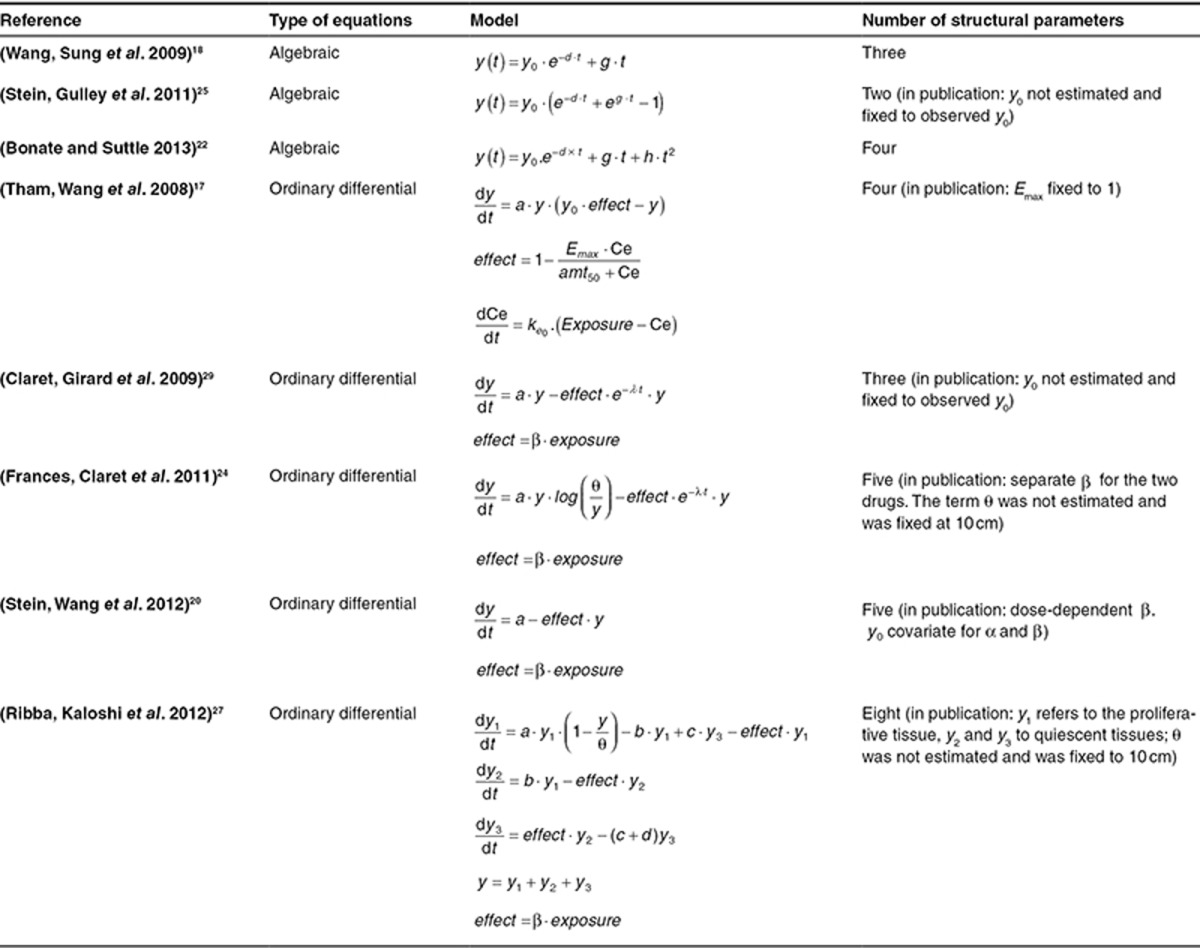
Summary of the mathematical equations and the complexity of the eight different models published for the analysis of tumor size dynamics in patients

**Table 2:**
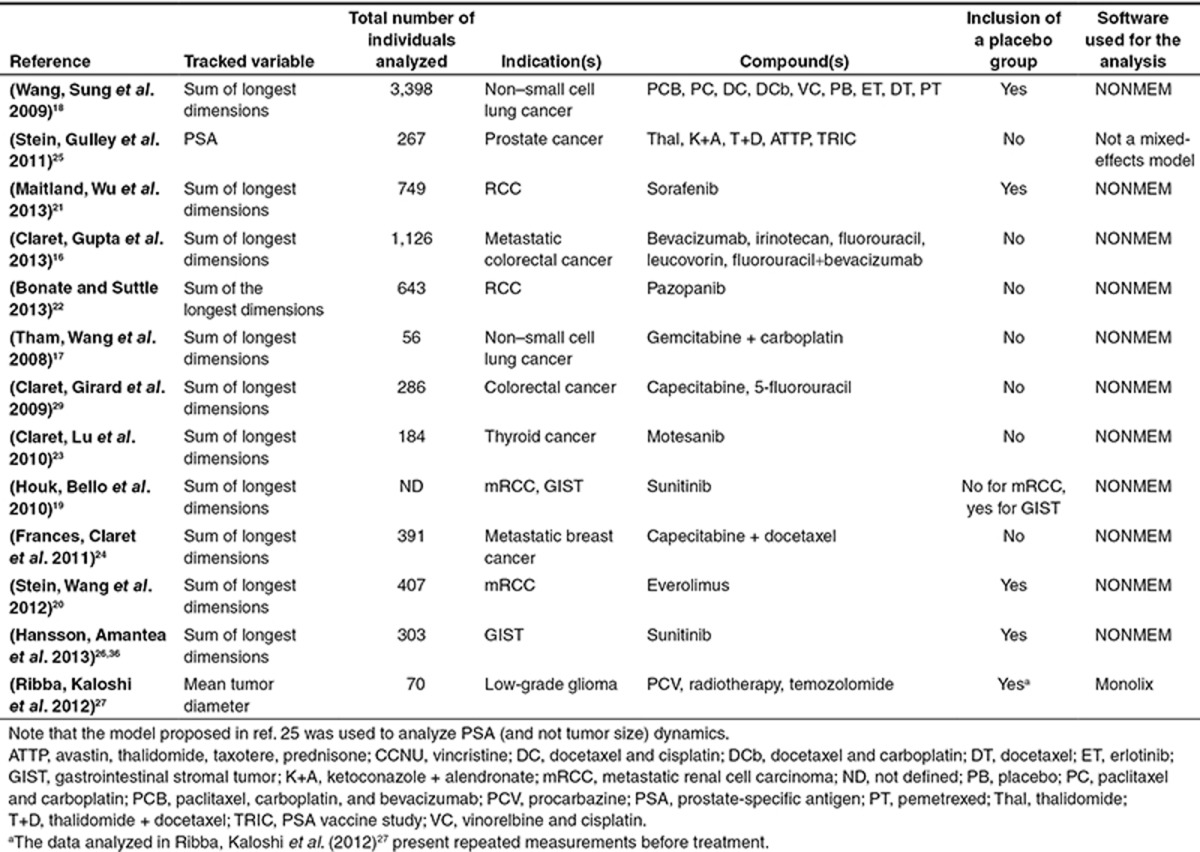
Main details of the 13 studies presenting analysis of tumor size dynamics in clinical settings
